# ﻿A new genus and species of minute litter bugs family Schizopteridae Reuter, 1891 from China (Hemiptera, Heteroptera, Dipsocoromorpha)

**DOI:** 10.3897/zookeys.1120.90086

**Published:** 2022-09-07

**Authors:** Jiu-Yang Luo, Qiang-Bang Gong, Qiang Xie

**Affiliations:** 1 State Key Laboratory of Biocontrol, Sun Yat-sen University, 135 Xingangxi Road, Guangzhou 510275, Guangdong, China; 2 School of Life Sciences, Sun Yat-sen University, 135 Xingangxi Road, Guangzhou 510275, Guangdong, China; 3 Yunnan Tongbiguan Provincial Nature Reserve, Dehong 678400, Yunnan, China

**Keywords:** *Cornonannusbui* gen. et sp. nov., Oriental Region, Schizopterinae, taxonomy, Yunnan Province

## Abstract

A new genus and species of Schizopteridae, *Cornonannusbui* gen. et sp. nov. is described from Yunnan Province, China. The new genus is closest to *Pachyplagia* Gross, 1951, *Ogeria*, Distant 1913, and *Kaimon* Hill, 2004 in morphology, but it can be distinguished from these genera by the male having a frontal process, the absence of pronotal collar, the distinct venation of forewing, a tarsi formula of 3-3-3, and the middle area of abdominal tergites I to VII with small, round tubercles. Photographs of the male habitus, head, thorax, abdomen, appendages, and genitalic structures, as well as scanning electron micrographs of the male head, thorax, abdomen, and genitalic structures and drawings of male genitalia, are provided. Moreover, a key to all known Chinese schizopterid genera is presented.

## ﻿Introduction

The Schizopteridae Reuter, 1891 are the most species-rich and widely distributed family of Dipsocoromorpha (the minute litter bugs), and the family currently contains two subfamilies, close to 60 genera, and about 355 species (Schuh and Weirauch 2020). Schizopterids include the smallest true bugs, ranging in size from 0.8 to 3.0 mm. Most of them have a compact and rotund appearance and are brown or nearly black. Additionally, they sometimes remarkably resemble certain beetles and members of the intertidal dwarf bugs, owing to their coloration and uniformly sclerotized, coleopteroid forewings (Štys 1995). Most of them are ground and litter dwelling, and some species are associated with low herbaceous vegetation, bark, subalpine heath, palm swamp, elevated bogs, moss, grass tussocks, and the nests of ants (Emsley 1969; Hill 1980, 1984, 1987, 1990a; Schuh and Weirauch 2020). Biological habits were observed and recorded by Emsley (1969).

Currently, five genera and 18 species of Schizopteridae are recorded from China (e.g. McAtee and Malloch 1925; Ren and Yang 1991; Ren and Zheng 1992; Rédei et al. 2012). Although the species of *Kokeshia* Miyamoto, 1960 were recently studied (Luo and Xie 2022), the species diversity of schizopterids from China is still poorly known. In this work, a new genus and species, *Cornonannusbui* gen. et sp. nov., is described, their detailed morphological structures are studied, and a key to all known schizopterid genera occurring in China is provided.

## ﻿Materials and methods

Specimens were collected in forests by light trapping. Specimens were preserved in 85% ethanol in the field.

External structures and genitalic structures were examined using a Zeiss Discovery V20 stereo microscope. Measurements were taken using a Zeiss Discovery V20 stereo microscope with the software ZEN v. 2.5 pro. Male head, thorax, abdomen, appendages, and genitalia were macerated in warm 10% potassium hydroxide solution (KOH). Habitus, head, thorax, abdomen, appendages, and genitalia were photographed using a Canon EOS 7D Mark II camera equipped with a tube lens and a Mitutoyo M Plan Apo 10× objective lens. Drawings of abdominal segment VIII, pygophore, parameres, and aedeagus were made using a camera lucida from an Olympus CX41 optical microscope under a 40× objective lens. Scanning electron micrographs of abdomen and genitalia of male were prepared using a Phenom Pro Scanning Electron Microscope. Maps were prepared using SimpleMappr (http://www.simplemappr.net/). The type series of the new species is deposited in the Museum of Biology, Sun Yat-sen University, Guangzhou, China (**SYSBM**).

The terminology used here mainly follows Hill (1990a, 1990b) and Knyshov et al. (2018), and abbreviations used in the text and figures are as follows: **a1–2** = antennal segments I to II; **ac** = axillary cord; **aed** = aedeagus; **ano** = anophore; anop = anophoric process; **ap** = adhesive pad; **at** = anal tube; **b** = buccula; **bc** = basal cell; **C** = costa; **cl** = clypeus; **cms** = cibarial muscle scar; **Cu** = cubitus; **dc** = discal cell; **epm1** = proepimeron; **epm2** = mesepimeron; **epm3** = metepimeron; **eps1** = proepisternum; **eps2** = mesoepisternum; **fe** = femur; **hp** = horn-like frontal process; **hpl3** = hyperpleural lobe of metathorax; **lb1–4** = labium segments I to IV; **lp** = left paramere; **lr** = labrum; **M** = media; **me3** = metendosternite; **mp** = maxillary plate; **ms** = mouthpart stylet; **pn** = pronotum; **pnt2** = mesopostnotum; **pnt3** = metapostnotum; **py** = pygophore; **px** = prosternal xiphus; **R** = radius; **rp** = right paramere; **sc2** = mesoscutum; **sc3** = metascutum; **Sc** = subcostal; **scc** = subcostal cell; **scl2** = mesoscutellum; **sp** = sternal plate of prothorax; **ss3** = metasternal spine; **s2–7** = sternites of abdominal segments II to VII; **tar1–3** = tarsal segments I to III; **tc** = trapezoidal cell; **tp** = triangular process; **t1–8** = tergites of abdominal segments I to VIII; **v** = vesica; **1An** = first anal vein; **2An** = second anal vein.

## ﻿Taxonomy

**Family Schizopteridae Reuter, 1891**

**Subfamily Schizopterinae Reuter, 1891**

### ﻿Genus Cornonannus gen. nov.

Taxon classificationAnimaliaHemipteraSchizopteridae

E7E8D75F-A852-54ED-BBC5-E1B2D9EF2A6E

https://zoobank.org/1AEACAED-23C0-4690-AB8E-5287B3E9E5D9

Figs 1 , 2 , 3 , 4 , 5 , 6 , 7 , 8 , 9

#### Type species.

Type species by present designation: *Cornonannusbui* sp. nov.

#### Diagnosis.

**Figure 1.**
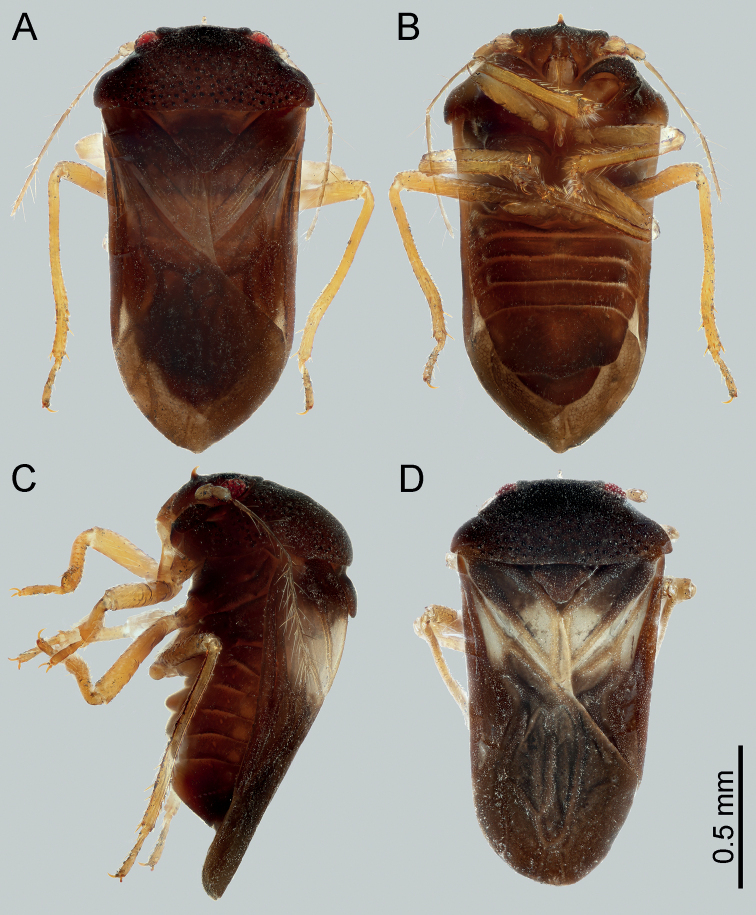
Habitus of *Cornonannusbui* gen. et sp. nov. **A–C** male holotype **A** in dorsal view **B** in ventral view **C** in lateral view **D** male paratype in dorsal view (in dry condition).

**Figure 2.**
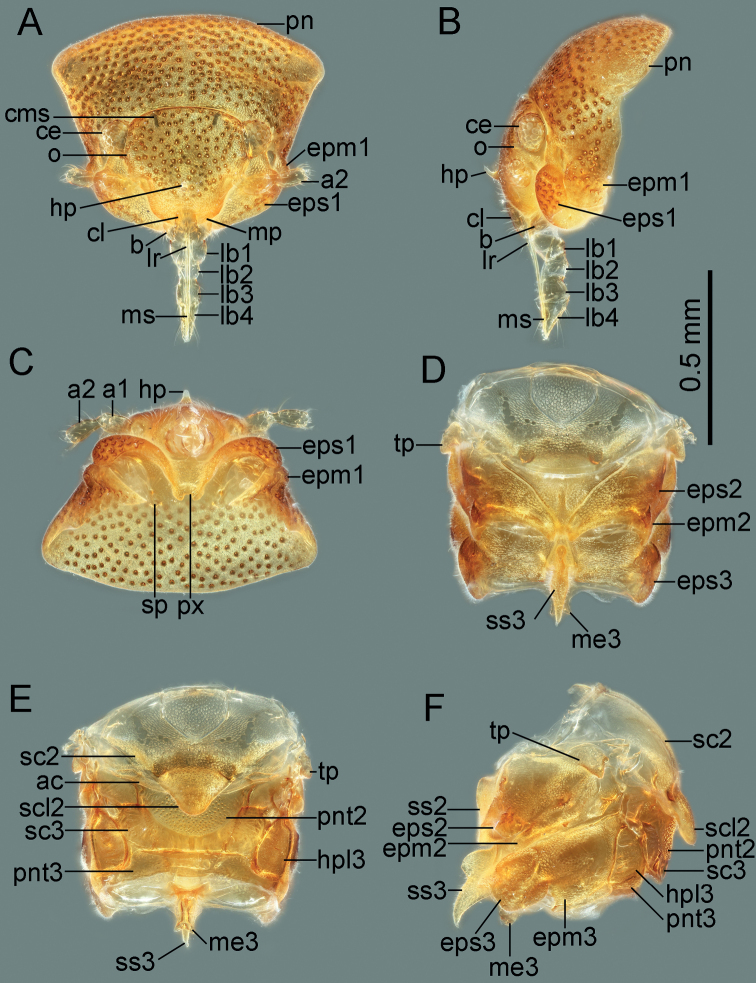
Head and thorax of *Cornonannusbui* gen. et sp. nov., male paratype **A–C** male head and prothorax **D–F** male pterothorax **A** in frontal view **B** in lateral view **C** in ventral view **D** in ventral view **E** in dorsal view **F** in lateral view.

**Figure 3.**
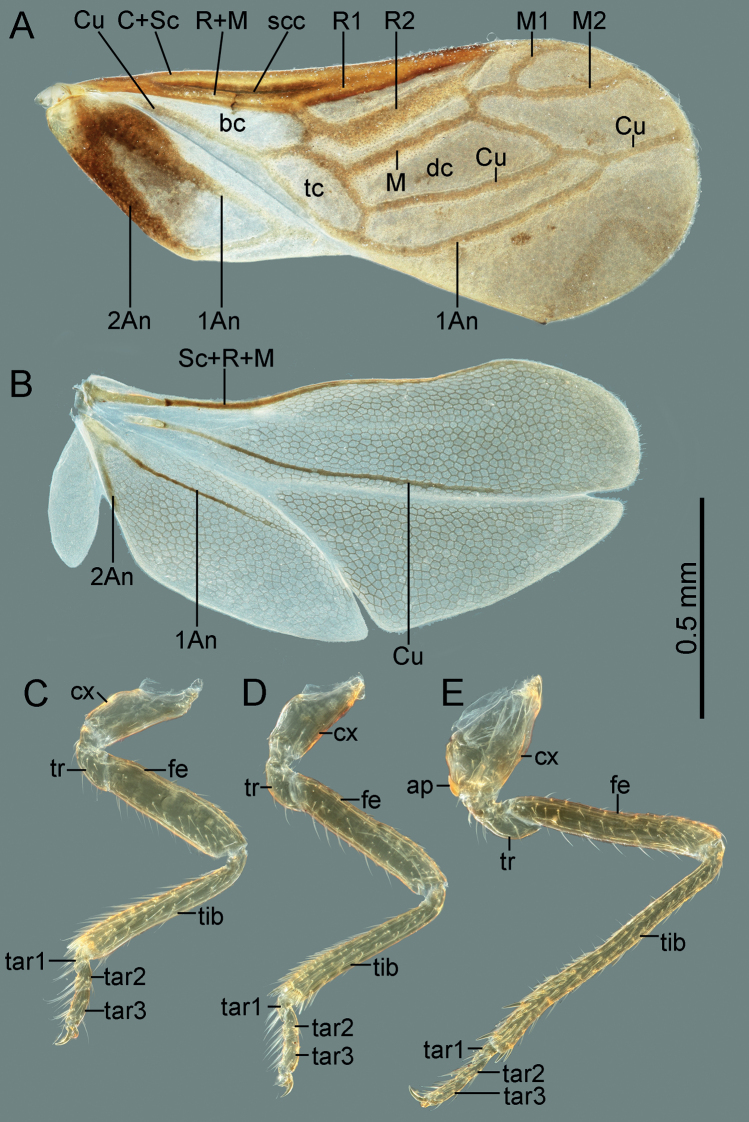
Wings and legs of *Cornonannusbui* gen. et sp. nov., male paratype **A** forewing in dorsal view **B** hindwing in lateral view **C** foreleg **D** middle leg **E** hind leg.

**Figure 4.**
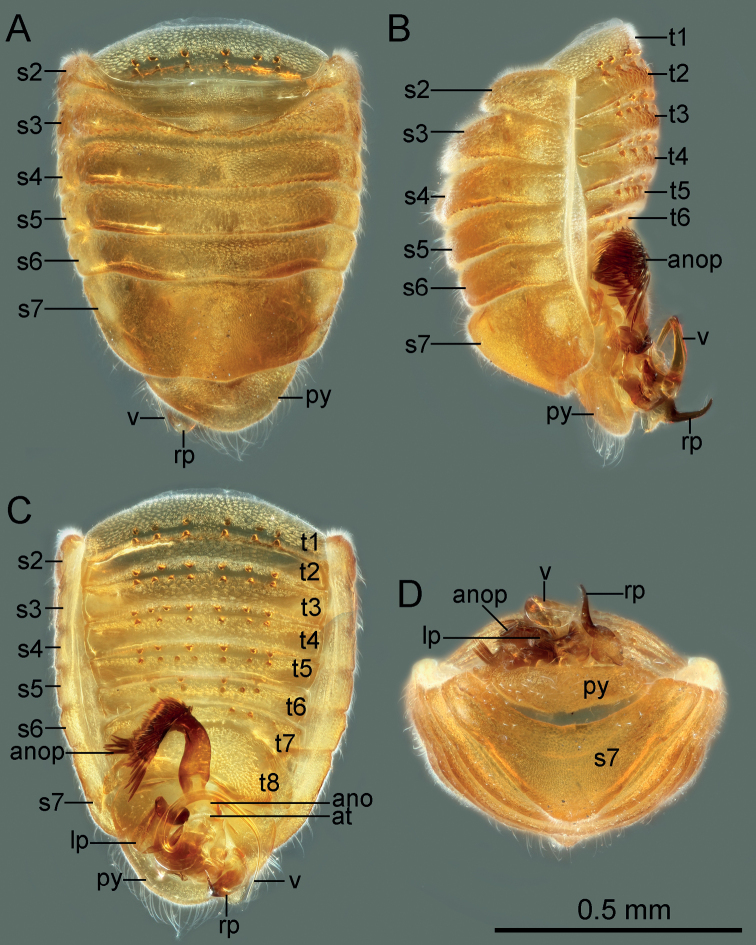
Abdomen of *Cornonannusbui* gen. et sp. nov., male paratype. **A** in dorsal view **B** in lateral view **C** in ventral view **D** in caudal view.

**Figure 5.**
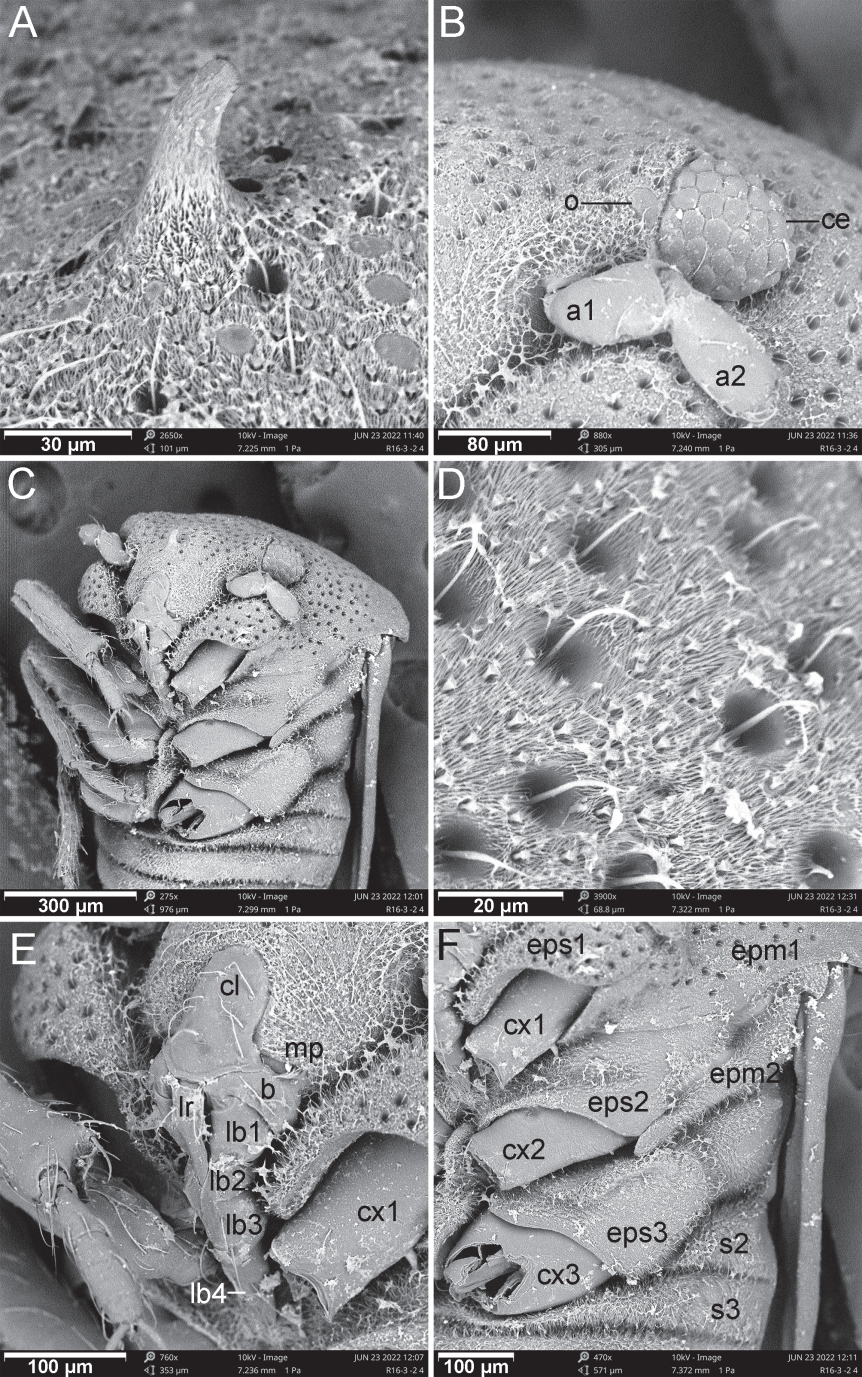
Scanning electron micrographs of *Cornonannusbui* gen. et sp. nov., male paratype in ventro-lateral view **A** frontal process **B** left eye and ocellus **C** head and thorax **D** microtrichia on head **E** mouth parts **F** pterothorax.

**Figure 6.**
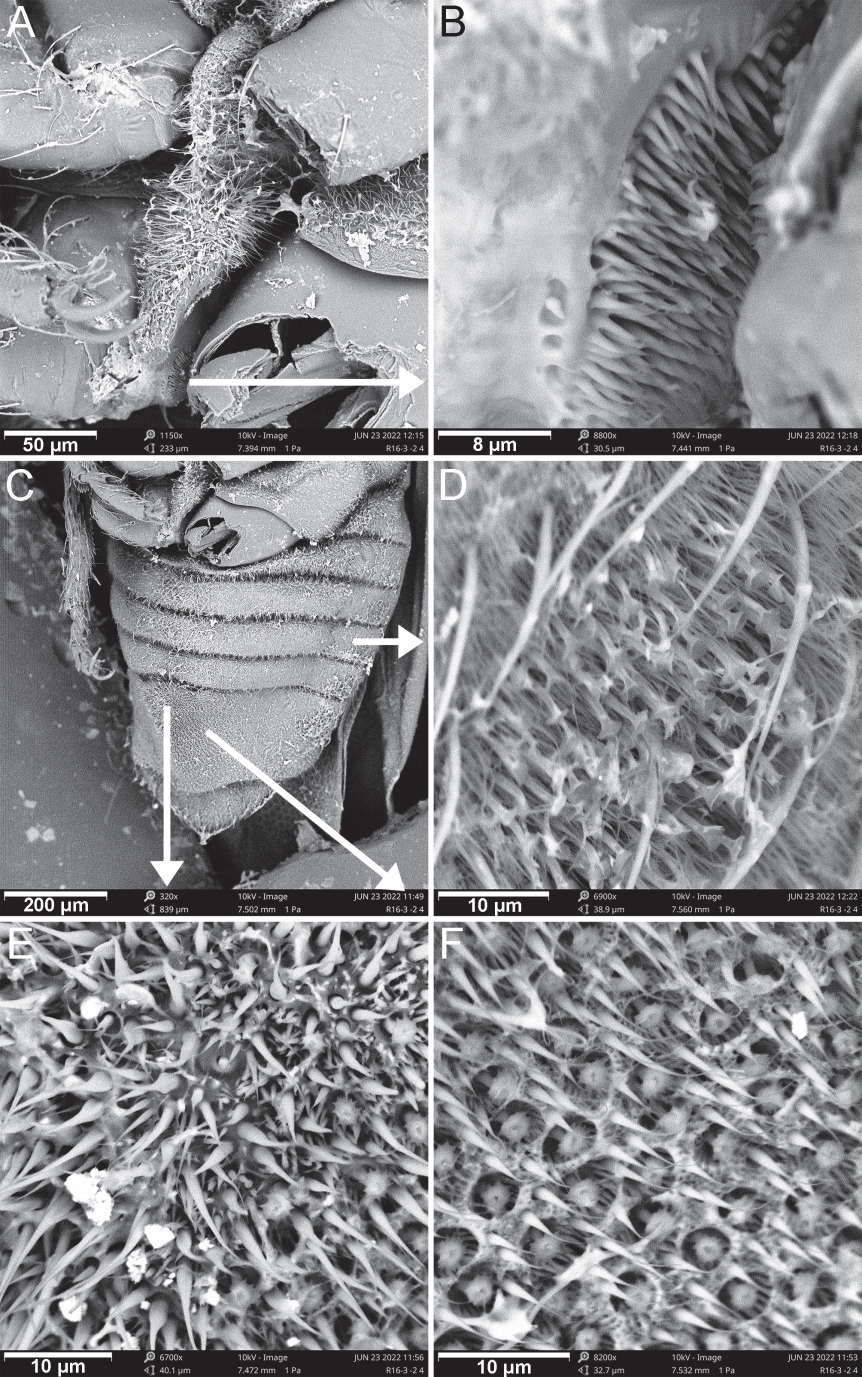
Scanning electron micrographs of *Cornonannusbui* gen. et sp. nov., male paratype in ventro-lateral view **A** metasternal spine **B** adhesive pad **C** abdomen **D** microtrichia on area near connexivum **E** middle region of basal sternite VII **F** middle region of sternite VII.

**Figure 7.**
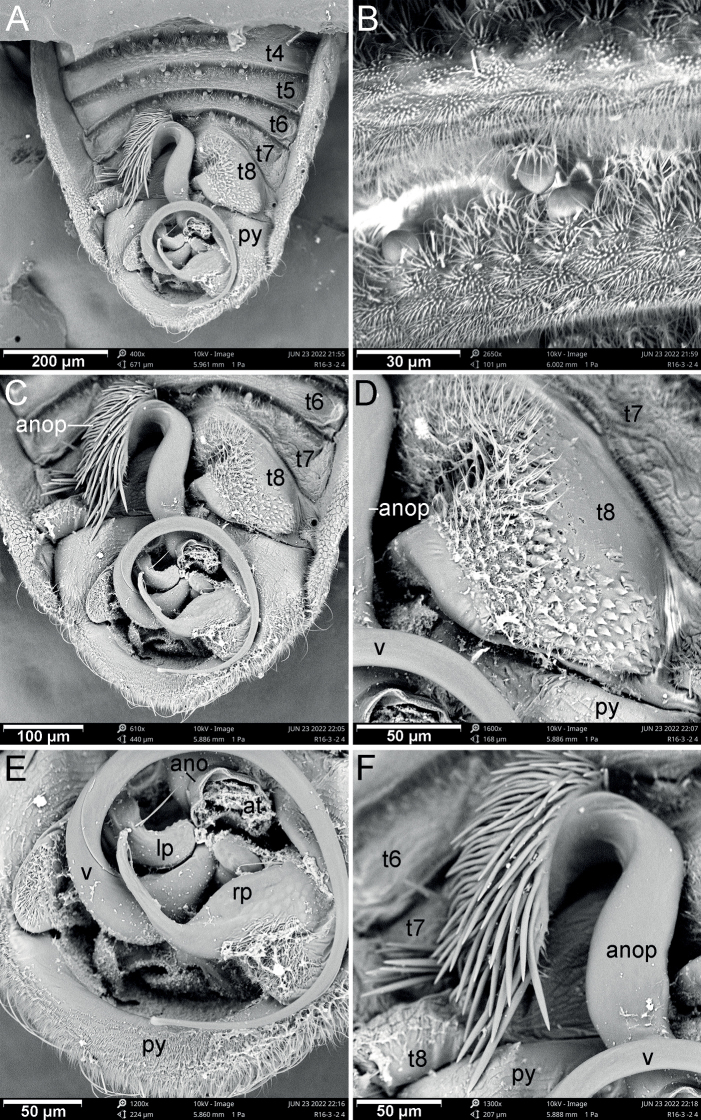
Scanning electron micrographs of *Cornonannusbui* gen. et sp. nov., male paratype in dorsal view **A** abdomen **B** groove and tubercles on tergite V **C** apex of abdomen **D** right region of tergite VIII **E** genitalia **F** apex of anophoric process.

The genus *Cornonannus* gen. nov. can be distinguished from the other genera of Schizopterinae by the following combined characteristics: 1) male with an upcurved horn-like frontal process on middle of head (Figs 1A–D, 2A–C); 2) pronotum without collar; 3) forewing without costal fracture (Fig. 3A); 4) forewing C+Sc, R1 distinctly thicker than other veins, R2 broad, about 2–3 times width of M, Cu or apical half of An (Fig. 3A); 5) tarsal formula 3-3-3 (Fig. 3C–E); 6) tergites I–VII of abdomen with transverse groove, tubercles on both sides of groove respectively or only on posterior side of groove (Fig. 4B, C); 7) male anophoric process large and curved (Fig. 4B, C).

#### Description.

Small (ca 1.5–1.6 mm), oblong and stout, forewing exceeding apex of abdomen (Fig. 1A–D). ***Coloration***: ground color brown to dark brown, compound eyes red to dark red, appendages yellowish brown to light brown, subapical of forewings with whitish area (Fig. 1A, D).

**Structure: *head*** strongly declivent, short in lateral view, with dense punctures; frons slightly convex, with frontal process in the middle (Figs 2A, C, 5A, C), and several frontal cibarial muscle scars; vertex with cibarial scars consists of five contiguous coarse pits arranged in a row on both sides (Fig. 2A); areas near inner margin of eyes convex; maxillary plate and buccula with semi-erect, long setae, clypeus with three pairs of semi-erect setae, and one central long seta; labrum with one pair of long setae, labial segment IV with two pairs of long setae (Figs 2A, B, 5C, E). Compound eye with about 30 ommatidia. Ocelli small, near inner margin of eyes, as large as about two ommatidia (Figs 2A, B, 5B). Antennae four-segmented, antennal segments I and II stout, subequal in length, with several semi-erect setae (Fig. 2A–C); segments III and IV slender, segment IV longer than III, both segments with very long, semi-erect setae (Fig. 1A–C). Labium four-segmented, reaching to middle of mesosternum, segment I thickest, segment IV longest and tapering (Fig. 1A, B). ***Thorax***: prothorax with relatively dense punctures as in head (Figs 2A, B, 5C–D). Pronotum near trapezoidal, declivent, without collar; callosite region with muscle scars consist by several pits (Fig. 2A); disk region convex; lateral margin sinuate, posterior margin slightly convex, middle near straight; proepisternal lobe inflated, almost reaching antennal insertions in lateral view (Fig. 2B). Proepimeral lobe wider than proepisternal lobe, subrectangular, posterior margin of proepimeron sinuate (Fig. 2B). Prosternum with wide longitudinal middle groove, apical portion of prosternal xiphus bilobate, sternal plate of prothorax trapezoidal, with straight posterior margin (Fig. 2C). Mesoscutum large; apex of mesoscutellum projecting posterodorsally in lateral view, base of mesoscutellum with a pair of pits (Fig. 2D–F); axillary cord sinuated; mesopostnotum with dense tubercles (Fig. 2E, F); mesoepisternal lobe large, mesepimeral lobe narrow, with tongue-shaped apex; upper area of mesopleura with a caudally directed triangular process (Fig. 2 D–F), mesopleura with groove along with middle coxal cleft, a deep concave located near the middle (Figs 2F, 5F); mesosternum with longitudinal mesosternal ridge (Fig. 2F). Metascutum transverse, curved; metapostnotum transverse; a deep groove between mesopleura and metapleural (Figs 2F, 5F). Metasternum with large keel-like metasternal spine, interlocking with notch between middle coxal cavities, basal portion of metasternal spine with round process, triangular apex (Figs 2F, 6A); apex of metendosternite bilobate (Fig. 2D–E). Forewings macropterous in males, female unknown; C+Sc and R thickened, with very narrow, slender subcostal cell; R2 distinctly wider than other veins; basal cell and trapezoidal discal cell slender; end of M subdivided into two branches (Fig. 3A). Hind wings large and subdivided into four lobes, jugal lobe small; Sc+R+M and Cu subequal in length, 1An about three times as long as 2An, m-cu absent, (Fig. 3B). Tarsal formula 3-3-3; apex of coxae, whole trochanters, femurs, tibiae, and tarsi with semi-erect setae (Fig. 3C–E). Arolia absent; pulvilli fine and straight. Inner apex of foretibiae protuberant, with a long seta (Fig. 5E); foretibiae bristle comb with about 10 setae; middle tibiae bristle comb with about 10 setae. Inner surface of hind coxae with adhesive pad (Figs 3E, 6A, B); distal half of hind tibiae slightly curved, apex of hind tibiae with four thick setae. ***Pregenital abdomen***: abdomen strong sclerotized and rigid; terga interlocking, sterna also interlocking; tergites I–VI, sternites II–VI almost symmetric, tergite VII, sternite VII slightly asymmetric, tergite VIII strongly asymmetric (Fig. 4A–C). Tergites I and II fused, with transverse groove in the middle, tubercles on both sides of groove respectively or only on posterior side of groove (Figs 4B, C, 7A, B). Middle area of sternites II and III less sclerotized, as sockets interlocking with metasternal plate; middle of sternite IV with longitudinal ridge; posterior margin of sternites VI and VII sinuated; sternite VII showing distinct sinistral asymmetry, sternites on both sides are approximately 90° from caudal view (Fig. 4D). Tergite VIII also showing sinistral asymmetry. ***Genitalia***: basal portion of pygophore near globular, overlapped by tergite VIII and sternite VII in repose; apical portion of pygophore flat, and exposed, with dense setae, asymmetrical. Anophore tubular, with anophoric process. Parameres strongly asymmetry; left paramere short, with a broad base, and a curved flattened distal projection; right paramere long, tapering, with a flat oval base provided with inner curved distal projection. Aedeagus complex, with a large, triangular basal plate; apical portion tubular, thin.

#### Etymology.

The generic name is derived from the Greek prefix “corn-” (horned or having horns or horn-like appendages) and the Greek root “nannus” (a dwarf). The gender is masculine.

### ﻿ Cornonannus bui

Taxon classificationAnimaliaHemipteraSchizopteridae

sp. nov.

47B7F191-DF6B-5A53-A250-BF1F0AE6B5A4

https://zoobank.org/079DE0DD-53EA-4F0F-BB1A-0290C7BF6044

Figs 1 , 2 , 3 , 4 , 5 , 6 , 7 , 8 , 9

#### Material.

***Holotype***: ♂, China, Yunnan Province, Dehong Prefecture, Yingjiang County, Sudian, Mengga River: 25°5'36"N, 97°51'27"E; ca 1420 m elev.; leg. Qiang Xie &Yue-Ran Wang; 2019-VIII-20 (SYSBM). ***Paratypes***: 4♂♂, same data as holotype (SYSBM).

#### Diagnosis.

Same as diagnosis of genus.

#### Description.

**Macropterous male**: small (1.47–1.64 mm), oblong and stout (Fig. 1A–D). ***Coloration***: ground color brown to dark brown, appendages yellowish brown to light brown, compound eyes dark red, ocelli light brown; punctures on head and prothorax black; distal half and about 2/3 of anterior margin of clavus, basal cell and base of trapezoidal cell of forewings whitish (Fig. 1A, D).

**Surface and vestiture**: head and prothorax with relatively dense setae, dense hair-like and end-enlarged microtrichia (Fig. 5A–B, D). Pterothorax with numerous tiny, oblong tubercles and dense microtrichia (Fig. 5F). Veins of forewings with sparse setae. Sterna of abdomen with sparse setae (Fig. 6C), dense microtrichia; area near connexivum with end-enlarged microtrichia (Fig. 6D). Middle region of basal sternite VII with stout-based microtrichia (Fig. 6E), middle region of sternite VII with dense small pits which with radial-ended microtrichia in the middle (Fig. 6F). Tergites of abdomen with tiny, oblong tubercles and dense microtrichia (Fig. 7A–B).

**Figure 8.**
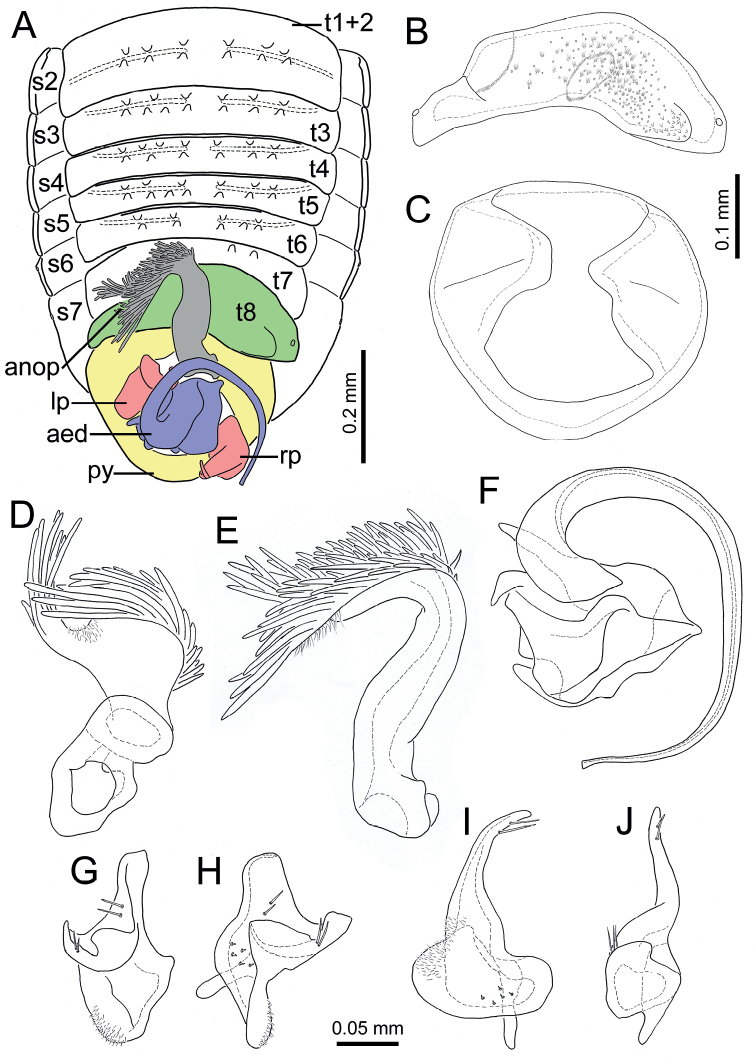
Drawings of *Cornonannusbui* gen. et sp. nov., male paratype **A** abdomen in dorsal view **B** tergite VIII in dorsal view **C** pygophore in dorsal view **D, E** two aspects of anophoric process **F** Aedeagus in dorsal view **G–H** two aspects of left paramere **I, J** two aspects of right paramere. Scale bars: 0.2 mm (**A**); 0.1 mm (**B, C**); 0.05 mm (**D–J**).

**Structure: *head*** short; wider than long; frons with up-curved frontal process in middle region (Figs 2A, C, 5A, C). Eyes relatively small, minimum width of vertex/maximum width of eye ca 3.82. Ocelli small, oval. Antennae four-segmented, antennal segment I and II stout, subequal in length, segment III and IV slender, segment IV longer than III, ratio of antennal segments I:II:III:IV = 1:1.05:3.45:4.2. Labium four-segmented, labial segment I thickest, segment IV longest and tapering, ratio of labial segments I:II:III:IV = 1.78:1:1.67:2.33. ***Thorax***: pronotum declivent, near trapezoidal, width about 1.83 times of middle length. Forewings C+Sc and R thicken, with very narrow, slender subcostal cell, R2 about three times the width of other veins (Fig. 3A). Tarsal formula 3-3-3. Forefemurs distinct thicker than middle and hind femurs. ***Pregenital abdomen***: abdomen strongly sclerotized, rigid; tergite I–VI, sternite II–VI almost symmetric, tergite VII, sternite VII slightly asymmetric, tergite VIII strongly asymmetric (Fig. 4A–C); tergites I and II fused, with transverse groove in the middle, and 6–7 round tubercles on both sides of groove respectively; tergites III–VII with transverse groove near basal margin, tergites III–V with 6–7 round tubercles on both sides of groove respectively, tergite VI with four or five round tubercles on both sides of groove respectively, tergite VII with four round tubercles only on posterior side of groove (Figs 4B, C, 7A, B). Tergite VIII also showing sinistral asymmetry, left area depressed which accommodating anophoric process (Fig. 8B). ***Genitalia***: anophore short tubular, with large anophoric process, curving to left; anophoric process with dense spiniform setae on apical region (Figs 7C, F, 8D, E). Left paramere with microtrichia at base, and oblong flattened distal projection which with two erect setae in dorsal surface; dorsum with hemispherical lobe, which with bilobate margin, two erect setae situated near the concave (Fig. 8G, H); distal projection of right paramere with two semi-erect setae on inner margin (Fig. 8I, J). Aedeagus complex, with a large, triangular basal plate; apical portion tubular, thin, protruding from pygophore about one coil (Fig. 8A, F).

**Female**: unknown.

#### Measurements

**(in mm; male holotype / male paratypes, *N* = 4).** Total body length 1.59 / 1.47–1.64; length of head 0.31 / 0.30–0.32, maximum width across eyes 0.51 / 0.48‒0.50, interocular distance 0.34 / 0.31‒0.34; length of antennal segment I 0.11 / 0.09–0.11, segment II 0.10 / 0.10‒0.11, segment III 0.37 / 0.32‒0.37, segment IV 0.44 / 0.40‒0.43; length of labial segment I 0.09 / 0.07‒0.09, segment II 0.04 / 0.04‒0.05, segment III 0.07 / 0.07‒0.08, segment IV 0.11 / 0.10‒0.11; middle length of pronotum 0.44 / 0.41‒0.46, humeral width 0.82 / 0.77‒0.81; length of forewing 1.32 / 1.27–1.47; length of fore femur 0.39 / 0.34–0.38, fore tibia 0.44 / 0.40‒0.43, fore tarsus 0.16 / 0.15–0.16; length of middle femur 0.43 / 0.38‒0.42, middle tibia 0.46 / 0.41‒0.45, middle tarsus 0.16 / 0.15‒0.17; length of hind femur 0.51 / 0.46–049, hind tibia 0.71 / 0.65–0.68, hind tarsus 0.19 / 0.18–0.19; greatest width of abdomen 0.66 / 0.61‒0.65.

#### Etymology.

The specific name is derived from and dedicated to Prof. Wenjun Bu (Institute of Entomology, Nankai University, China), recognizing his contributions to the study of taxonomy, phylogeography and biogeography of Heteroptera, on the occasion of his 60^th^ birthday.

#### Distribution.

**Figure 9.**
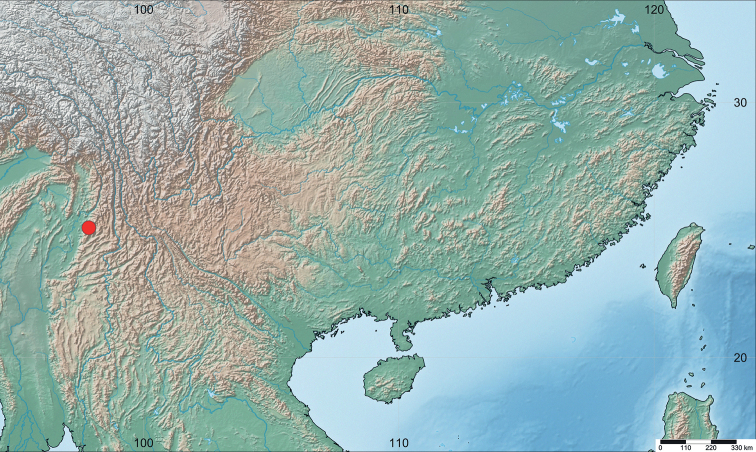
Distribution map of *Cornonannusbui* gen. et sp. nov.

Known only from the type locality, Yingjiang County, Yunnan Province, China (Fig. 9).

### ﻿Key to the known genera of Schizopteridae Reuter, 1891 from China

**Table:** 

1	Eyes exceedingly large, broadly overlapping anterolateral margins of pronotum as in Hill (2013: fig. 3); clypeus with 4 or 5 macrosetae as in Hill (2013: fig. 4)	***Hypselosoma* Wygodzinsky, 1959**
‒	Eyes of moderate size, overlapping at most anterolateral angles of pronotum; clypeus without macrosetae	**2**
2	Labium 3-segmented as in Wygodzinsky (1950: figs 85–87); tergite VIII of male with a large bladder-like appendage as in Wygodzinsky (1950: figs 104–105, 107, 119–122)	***Dundonannus* Wygodzinsky, 1950**
‒	Labium 4-segmented; tergite VIII of male without large bladder-like appendage	**3**
3	Forewing with costal fracture as in Luo and Xie (2022: figs 26, 27)	***Kokeshia* Miyamoto, 1960**
‒	Forewing without costal fracture	**4**
4	S+Sc of forewing with a row of tiny pit at basal area as in Ren and Yang (1991: fig. 4)	***Sculptocoris* Ren & Yang, 1991**
‒	S+Sc of forewing without a row of tiny pit at basal area	**5**
5	Tarsal formula 2-2-3 in male; pronotal collar present as in Hill (1990a: figs 1, 2, 5, 6, 30, 40); head of male without frontal process as in Hill (1990a: figs 1, 2, 5, 6, 30, 40)	***Pachyplagia* Gross, 1951**
‒	Tarsal formula 3-3-3 in male (Fig. C–E); pronotal collar absent (Fig. 2A, B); head of male with a horn-like frontal process (Figs 2A–C, 5A, C)	***Cornonannus* gen. nov.**

## ﻿Discussion

The genus *Cornonannus* gen. nov. is morphologically most similar to *Pachyplagia* Gross, 1951, *Ogeria*, Distant 1913, and *Kaimon* Hill, 2004, but it also has significant differences from these three genera (Table 1). Until now, special organs on the head of schizopterids has been thought to be rare. Except for the new genus and species, the other cases are as follows: male with bilobed frontal process, a synapomorphy of the genus *Voragocoris* Weirauch, 2012 (Weirauch 2012); male vertex of *Kaimon* usually with conspicuous pit or U-shaped groove and lobe (Hill 2004); some *Membracioides* species with vertex organ (Knyshov et al. 2019); and several *Hypselosoma* species with male labral organ.

**Table 1.** Comparision of *Cornonannus* gen. nov., *Ogeria* Distant 1913, *Pachyplagia* Gross, 1951, and *Kaimon* Hill, 2004 (partly modified from Hill 2004.

Character	*Pachyplagia*	*Cornonannus*	*Ogeria*	*Kaimon*
Body length	1.33–1.51mm	1.47–1.64mm	0.79–1.33mm	0.76–1.04mm
Head peculiar structure	absent	frons with process	absent	sometimes vertex with a pit or U-shaped groove and lobe
Frons cibarial muscle scars	discrete from vertex group		not evident	concurvilinear with vertex group
Form of vertex cibarial scars	deep furrow × 2	5 coarse pits × 2	4 coarse pits × 2	several fine pits × 2
Pronotal collar	present	absent	present	absent
Male tarsal formula	2-2-3	3-3-3		
Male fore- and middle tarsi	slender			incrassate
Forewing C+SC of macropterous	greatly inflated	normal	sometimes inflated	normal
Mesosternal keel	small		medium	large
Posterior margin of pygophore	convex			emarginate
Carinulation of male dorsum	tergites I–VI	tergites I–VII	tergites I–VIII	tergites I–VII
Length of carinulae on tergite III–VI	half tergite	half to three-quarter tergite	full tergite	third to half tergite
Carinulation form	long tubercles	round tubercles	contigous carinae	
Left paramere form	short, oblong			long, slender
Left paramere basal digit	present			absent
Right paramere form	long, slender		short, tapering	very short, trilobate
Anophoric appendage	present		absent	

Biology of the new species is poorly known, but females of *Cornonannusbui* sp. nov. were not found with the males. Similar to its allies, females of this species are speculated to have brachypterous or micropterous forewings. As for body color, it is not uncommon for the color pattern of forewings of *C.bui* sp. nov., similar pattern also appear in species of *Corixidea* Reuter, 1891, *Ommatides* Uhler, 1894, *Chinannus* Wygodzinsky, 1948, *Pachyplagia* Gross, 1951, *Voragocoris*, *Hypselosomops* Hoey-Chamberlain & Weirauch, 2016, and *Perittonannus* Weirauch, Knyshov & Hoey-Chamberlain, 2020. Considering that this color pattern generally occurs in both sexes, it may be related to cognate recognition.

## Supplementary Material

XML Treatment for Cornonannus

XML Treatment for Cornonannus bui
